# ERα36, a variant of estrogen receptor α, is predominantly localized in mitochondria of human uterine smooth muscle and leiomyoma cells

**DOI:** 10.1371/journal.pone.0186078

**Published:** 2017-10-11

**Authors:** Yitang Yan, Linda Yu, Lysandra Castro, Darlene Dixon

**Affiliations:** Molecular Pathogenesis Group, National Toxicology Program Laboratory (NTPL), National Toxicology Program, National Institute of Environmental Health Sciences (NIEHS), National Institutes of Health (NIH), Research Triangle Park, North Carolina, United States of America; University of PECS Medical School, HUNGARY

## Abstract

ERα36 is a naturally occurring, membrane-associated, isoform of estrogen receptor α. The expression of ERα36 is due to alternative splicing and different promoter usage. ERα36 is a dominant-negative effector of ERα66-mediated transactivational activities and has the potential to trigger membrane-initiated mitogenic, nongenomic, estrogen signaling; however, the subcellular localization of ERα36 remains controversial. To determine the cellular localization of ERα36 in estrogen-responsive human uterine smooth muscle (ht-UtSMC) and leiomyoma (fibroid; ht-UtLM) cells, we conducted systematic confocal microscopy and subcellular fractionation analysis using ERα36 antibodies. With Image J colocalizaton analysis plugin, confocal images were analyzed to obtain a Pearson’s Correlation Coefficient (PCC) to quantify signal colocalization of ERα36 with mitochondrial, endoplasmic reticulum, and cytoskeletal components in both cell lines. When cells were double-stained with an ERα36 antibody and a mitochondrial-specific dye, MitoTracker, the PCC for the two channel signals were both greater than 0.75, indicating strong correlation between ERα36 and mitochondrial signals in the two cell lines. A blocking peptide competition assay confirmed that the mitochondria-associated ERα36 signal detected by confocal analysis was specific for ERα36. In contrast, confocal images double-stained with an ERα36 antibody and endoplasmic reticulum or cytoskeletal markers, had PCCs that were all less than 0.4, indicating no or very weak signal correlation. Fractionation studies showed that ERα36 existed predominantly in membrane fractions, with minimal or undetected amounts in the cytosol, nuclear, chromatin, and cytoskeletal fractions. With isolated mitochondrial preparations, we confirmed that a known mitochondrial protein, prohibitin, was present in mitochondria, and by co-immunoprecipitation analysis that ERα36 was associated with prohibitin in ht-UtLM cells. The distinctive colocalization pattern of ERα36 with mitochondria in ht-UtSMC and ht-UtLM cells, and the association of ERα36 with a mitochondrial-specific protein suggest that ERα36 is localized primarily in mitochondria and may play a pivotal role in non-genomic signaling and mitochondrial functions.

## Introduction

Estrogen receptors belong to the nuclear receptor superfamily, whose members include estrogen receptor alpha (ERα), estrogen receptor beta (ERβ) and, estrogen-related receptors (ERRα, β, and γ) [1]. Estrogens control a variety of physiological and disease processes, notably reproduction, secondary sex characteristics, bone remodeling, and gynecologic cancer development. Estrogen’s effects can be transduced through canonical ERα (ERα66), or ERβ. Each estrogen receptor classically functions through direct binding with a specific ligand, such as estrogen (17-β estradiol) or a phytoestrogen, like genistein. However, other molecular pathways such as nongenomic or ligand independent growth factor signaling have been described for classical estrogen receptor regulation [2].

**Fig 1:**
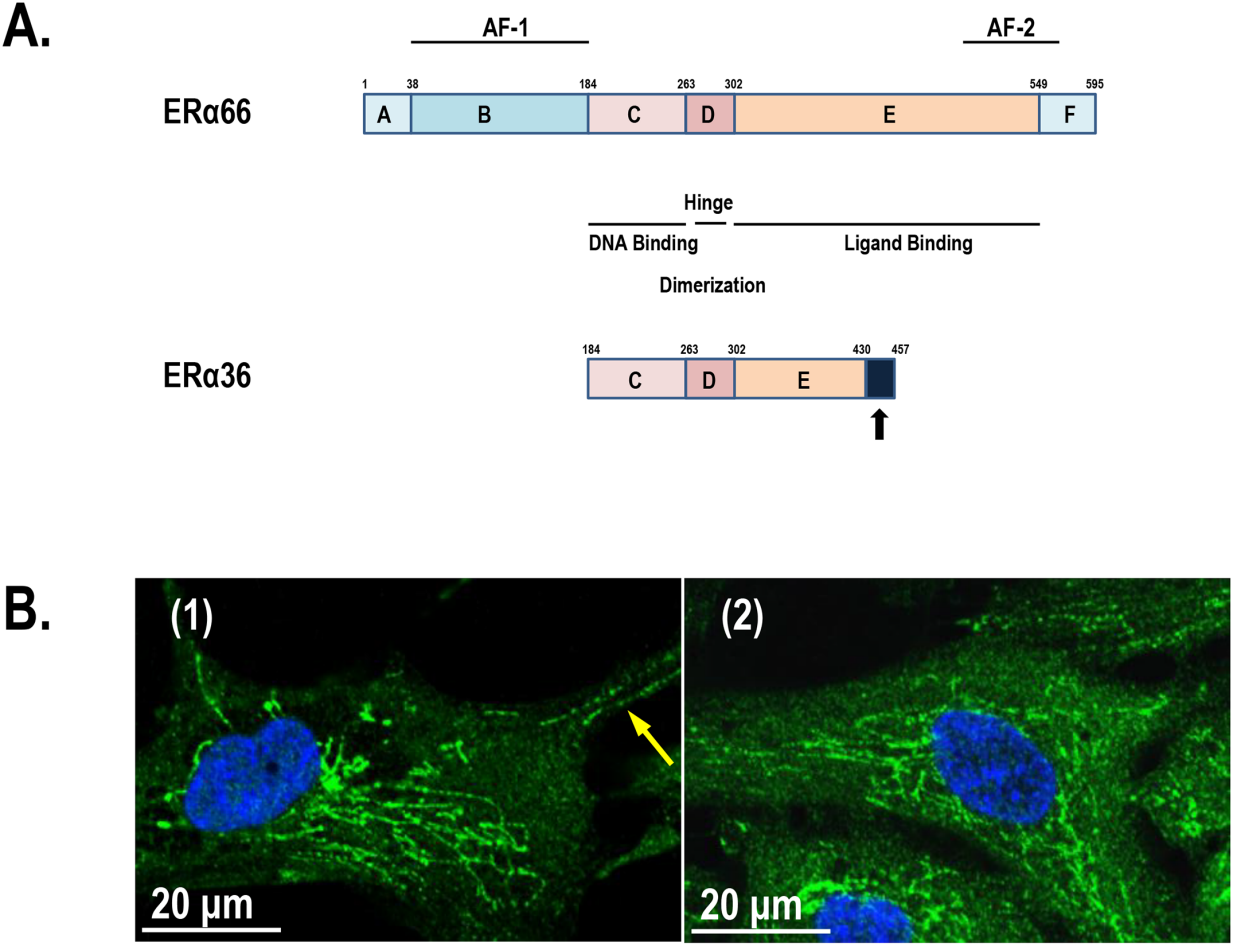
Comparison of ERα36 and ERα66 domain structures and subcellular ERα36 staining patterns in human uterine smooth muscle (ht-UtSMC) and leiomyoma cells (ht-UtLM). (A) The top structure shows six conserved domains of ERα66 (labeled A-F), the amino acid sequence numbers, and the transactivation function domains (AF-1 and AF-2). The function of each domain is indicated. In the domain structure of ERα36, the last unique 27 amino acids of ERα36 are indicated as a filled box. The diagram is drawn proportionally to domain size. The drawing is based on the publication by Wang et. al., 2005 and GenBank entry CAE45969.1. (B) In ht-UtSMC cells (1), distinctive ERα36 signals were detected along the plasma membrane as shown by the arrow, but mostly localized within a robust intracytoplasmic network. In ht-UtLM cells (2), the ERα36 signals were detected mostly in a robust network structure within the cytoplasm. The cell samples were stained with ERα36 antibody and DAPI.

Recently, a novel splice variant of the human ERα, named ERα36, was cloned from human placenta mRNA. ERα36 lacks both transactivation function domains, AF-1 and AF-2, of the full-length ERα (ERα66), and possesses an intact DNA-binding domain and a truncated ligand-binding and a partial dimerization domain [3] (Fig 1A). ERα36 was reported to be predominantly associated with the plasma membrane where it was found to transduce both estrogen- and antiestrogen-dependent activation of the mitogen-activated protein kinase/extracellular signal-regulated kinase (MAPK) signaling pathway. ERα36 lacks intrinsic transcriptional activity and mainly mediates non-genomic estrogen signaling [4]. ERα36 is expressed in both ER-positive and ER-negative breast cancer; however, ERα36 expression is more abundant in ER-negative breast cancer that constitutes ~30% of all breast cancers diagnosed in women in the US and is generally a more aggressive cancer that, typically lacks wild-type ERα (ERα66)-positive cells [3]. ERα36 is predicted to serve as a dominant-negative effector of ERα and ERβ estrogen-mediated genomic signaling and has the capacity to trigger membrane-initiated mitogenic estrogen signaling. ERα36 contributes to the resistance of breast cancer to selective estrogen receptor modulator (SERM) therapy, i.e., tamoxifen treatment. Due to the high levels of ERα36 expression, ERα-negative breast cancer cells can maintain estrogen mitogenic signaling in the absence of wild-type ERα66, which may explain the acquired tamoxifen resistance [5]. ERα36 may serve as a target for treating ER-negative breast cancers [5] and for the treatment of breast cancers with acquired tamoxifen resistance [6]. Additionally, ERα36 may be important in regulating the normal estrous cycle. In the hamster ovary, ERα36 expression was upregulated during estrus, and the gonadotropin surge had a direct effect on ERα36 expression [7].

Despite the fact that ERα36 mediates important roles in non-genomic signaling in cancer growth, acquired tamoxifen resistance and normal estrous cycling, the exact subcellular localization of ERα36 is controversial. ERα36 was reported to be predominantly localized in the plasma membrane of both ERα and androgen receptor negative endometrial cancer (Hec1A) cells [8]. It has been shown in triple negative breast cancer cells that ERα36 is expressed in a diffuse, intracellular, and linear or dotted membranous pattern [9]. The receptor was also reported to exist in the Golgi apparatus and the nuclear membrane [9]. In seminoma Tcam-2 cells, ERα36 was associated with cytoplasmic filamentous structures beneath the plasma membrane that were determined to be actin microfilaments by immunogold labeling. The authors concluded that the colocalization with cytoskeletal microfilaments suggests a role of ERα36 in cell motility [10]. To date, the subcellular localization of ERα36 remains controversial in the literature and appears to vary between cell types [8–10]. Most of the previous research on ERα36 expression, localization and function has been done in cancer cells, and has focused mainly on breast cancer; however, it has also been studied in endometrial, gastric, colon and seminoma cancer cells [11].

Uterine fibroids (leiomyomas) are one of the most common hormonally-responsive benign tumors affecting women of reproductive-age [12]. Although the exact etiology of uterine leiomyomas remains unclear, the fact that the disease develops during the reproductive years and regresses after menopause indicates that fibroids are hormonally regulated. In order to understand the role of ERα36 in the pathogenesis of fibroids, we thought it important to identify the exact subcellular localization of ERα36 in uterine smooth muscle and leiomyoma cells.

## Materials and methods

### Cell cultures

Human uterine leiomyoma cell line (ht-UtLM) and normal uterine smooth muscle cell line (ht-UtSMC), immortalized via retroviral transfection (pLX1N vector, Clonetech Laboratory, Inc.) of human telomerase, were generated by our laboratory [13]. G418 Sulfate (Geneticin^®^ Selective antibiotic, Life Technologies Cat# 10131–035) was added to ht-UtLM and ht-UtSMCs cell culture media at the concentration of 1.0 μl/ml and 1.5 μl/ml, respectively. Human ovarian cancer (BG-1), breast cancer (MCF-7), endometrial cancer (Ishikawa), and vulvar leiomyosarcoma (SK-LMS-1) cell lines were all cultured in DMEM medium supplemented with 10% FBS. All of the cultures were kept in a standard tissue culture incubator at 37°C with 5% CO_2_.

### Confocal fluorescence microscopy

The cells were grown in glass bottom microwell dishes (MatTek Corporation, Ashland, MA, USA, Part No# P35G-1.5-14-C). Approximately 50,000 cells were introduced to each dish, and grown for 3 days at 37°C in a CO_2_ incubator to reach 80% confluency. The cells were then fixed in cold methanol (-20°C) for 5 min on ice. The ERα36 antibody used for confocal analysis was kindly provided by Prof. Zhao-Yi Wang (Creighton University Medical center, Omaha, Nebraska). The cells were incubated with the ERα36 antibody (1:100 dilution) at 4°C overnight, and followed by incubation with Alexa Fluor^®^ 488 goat anti-rabbit IgG (H+L) antibody (1:3000 dilution, Molecular Probes, Eugene, OR, USA, Cat# A11008) at room temperature for 1 hour. The cells were then counterstained with 100 ng/mL 4,6-diamidino-2-phenylindole (DAPI, Molecular Probes, Cat# D1306) for 30 min. Confocal images were taken on a Zeiss LSM710-UV Confocal Microscope (Carl Zeiss, Oberkochen, Germany) using a Plan-Apochromat 63X/1.40 oil DIC M27 objective.

SelectFX^®^ Alexa Fluor^®^ 488 Endoplasmic Reticulum Labeling Kit (Invitrogen, Carlsbad, CA, USA, Cat# S34253) was used for colocalization studies. In this kit, Protein Disulfide Isomerase (PDI) antibody was used to specifically recognize endoplasmic reticulum. MitoTracker^®^ Deep Red FM (Invitrogen Cat# M22426) was used to visualize mitochondria. Fluorescently-labeled rhodamine phalloidin (Invitrogen Cat# R415) was used to visualize linear actin microfilament network, F-actin. All were used according to the manufacturer’s instructions.

### Quantitative colocalization analysis

The Image J Just Another Colocalization Plugin (JACoP) was applied for quantitative colocalization analyses. Pearson’s correlation coefficient was calculated to provide an estimate of the “goodness” of the linear approximation. Cross-correlation analysis was performed to statistically evaluate colocalization [14].

### Peptide competition assay for ERα36 antibody

Human ERα36 blocking peptide corresponding to the C-terminus unique sequence of ERα36 was purchased from Alpha Diagnostic International (San Antonio, TX, Cat# ERA361-P). The ERα36 antibody was diluted in blocking buffer (1:100 dilution) and a five-times excess of blocking peptide by weight was added to the antibody solution. The mixture was incubated with agitation overnight at 4°C. The confocal staining procedure as described above was done with neutralized and unblocked antibody.

### Subcellular protein fractionation and mitochondrial isolation

The Subcellular Protein Fractionation Kit for Cultured Cells (Pierce Biotechnology, Waltham, MA, USA, Cat# 78840) was used to prepare the various subcellular protein extracts according to the manufacturer’s instructions. The ht-UtSMC and ht-UtLM cells were cultured to approximately 90% confluency and were harvested and fractionated. The mitochondrial isolation kit for cultured cells (Pierce Biotechnology, Cat# 89874) was purchased for preparing mitochondrial fractions, and extracts were prepared according to the manufacturer’s instructions.

### Western blot analysis

The ERα36 antibody was purchased from Cell Applications (San Diego, CA, USA, Cat# CY-1109). This ERα36 antibody was diluted 1:10,000 in blocking buffer for western blot analyses. Protein concentrations were measured with a Pierce^™^ BCR Protein Assay kit (Thermo Scientific, Waltham, MA, USA, Cat# 23225) and SpectraMax M5 multi-mode microplate reader (Molecular Devices, Sunnyvale, CA, USA). Protein subcellular fractionations (10 μg per fraction) were analyzed with antibodies targeting specific antigens for various cellular compartments. The following primary antibodies anti-HSP90 (C45G5) Rabbit mAb (Cell Signaling, Danvers, MA, USA, Cat# 4877), anti-EGFR (1005) Rabbit (Santa Cruz Biotechnology, Cat# sc-03), anti-SP1 (D4C3) Rabbit mAb (Cell Signaling, Cat# 9389), anti-Histone H3 Rabbit (Cell Signaling, Cat#9715S), and anti-Vimentin (R28) Rabbit (Cell Signaling, Cat#3932) were used to analyze cytoplasmic, membrane, nuclear, chromatin-bound, and cytoskeletal fractions, respectively. The mitochondrial fractions were analyzed with Mortalin antibody (Novus Biologicals, Littleton, CO, USA, Cat# NBP 1–47801 mouse mAb), Prohibitin antibody (Thermo Scientific, Cat# MS-261-P1, mouse mAb), and Src antibody (Cell Signaling, Cat# 2123S, Rabbit mAb) to examine the purity of the mitochondrial preparation. The primary antibodies were diluted at 1:1000 with a blocking buffer (5% BSA in 1X TBST), and the nitrocellulose membrane blots incubated overnight. For secondary antibody incubations, HRP-conjugated ECL^™^ Anti-Rabbit IgG from donkey (GE Healthcare Life Sciences, Chicago, IL, USA, Cat# NA934V) or HRP-conjugated ECL^™^ Anti-Mouse IgG from sheep (GE Healthcare Life Sciences, Cat# NA931V) was diluted at 1:5000 and incubated on the blots for 1 hour. The protein blots were subsequently detected with Amersham ECL^™^ western Blotting Detection Reagents (GE Healthcare Life Sciences, Cat# RPN 2106).

### Co-immunoprecipitation analysis

The ht-UtLM cell culture was maintained in DMEM medium without phenol red, supplemented with 10% dextran-charcoal stripped FBS for 24 hrs, and then treated with 10^−8^ M and 10^−6^ M 17β-Estradiol (E_2_) (Steraloids, Newport, RI, USA, Cat# E0950-000) or ethanol vehicle control for 24 hrs in the same DMEM medium. The cell cultures were washed twice with ice-cold 1X PBS and lysed with lysis buffer (150 mM NaCl, 0.5% Triton X-100, 0.05% Na deoxycholate, 4% glycerol, 1 mM DTT, 10 mM Tris-HCl, pH 7.4) supplemented with protease inhibitor cocktail tablet (Roche Life Science, Indianapolis, IN, Cat# 11836153001). The 50 μl of Dynabeads^®^ Protein G (Thermo Fisher Scientific, Cat# 10004D) was incubated with 4 μg ERα36 antibody (Cell Applications, Cat# CY1109) for 10 min at room temperature. The Dynabeads-Ab complex was resuspended with cell lysate and incubated on a rotation rocker at 4°C overnight. The Dynabeads-Ab-Antigen complexes were washed three times using 200 μl washing buffer (1x PBS, pH 7.4), eluted with 20 μl elution buffer (50 mM Glycine, pH 2.8), separated on SDS-PAGE, and analyzed by western blotting with ERα36 (Cell Applications, Cat# CY1109) and prohibitin (Thermo Fisher Scientific, Cat# MS-261-P1) antibodies.

## Results

### Subcellular localization of ERa36 in ht-UtSMC and ht-UtLM cells

To investigate the role of ERα36 (Fig 1A) in non-genomic mitogenic signaling in human uterine smooth muscle physiology and fibroid pathogenesis, it is imperative to know the exact subcellular compartment where ERα36 is located. When ht-UtSMC and ht-UtLM cells were grown at low density in glass-bottom microwell dishes, and stained with ERα36 antibody, a unique staining pattern was observed (Fig 1B). In ht-UtSMC cells, the ERα36 antigen signals were confined mostly to distinctive network structures within the cytoplasm. The positive ERα36 signals were also detected along the plasma membrane as indicated by the arrow (Fig 1B). There were no detectable ERα36 signals or very minimal signals in the nuclei of both cell lines.

**Fig 2:**
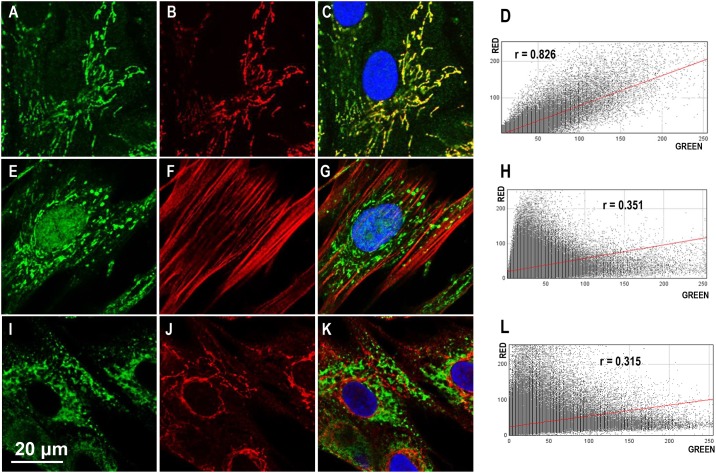
Colocalization analysis of ERα36 with mitochondria, F-actin or endoplasmic reticulum in ht-UtSMC cells. Top row: A. ERα36 signal channel, B. MitoTracker signal, C. Merged image, D. Scatter plot; Middle row: E. ERα36 signal channel, F. F-actin dye signal, G. Merged image, H. Scatter plot; Bottom row: I. PDI signal channel, J. ERα36 signal, K. Merged image, L. Scatter plot. In the scatter plot, the symbol r represents the Pearson’s correlation coefficient. Scale bar = 20 μm for all images.

To determine the identity of the unique subcellular structures recognized by ERα36 antibody, we colocalized ERα36 signal with dyes or antibodies specific for mitochondria, F-actin and endoplasmic reticulum. As indicted in Fig 2A–2C, ht-UtSMC cells were double-stained with ERα36 antibody (green signal) and MitoTracker Deep Red FM (red signal). Confocal evaluation showed that both reagents stained similar network structures within the cells. To quantitatively assess if the green and red signals were colocalized, plugin JACoP was used. As calculated, the Pearson's Coefficient r was equal to 0.826 (Fig 2D). Additionally, to determine whether the observed colocalization represented coincidental overlap of signals, Cross-Correlation Function (CCF) analyses were applied, where the green image was shifted horizontally in both directions relative to red image, and the Pearson’s coefficient was plotted as the function of pixel shift [14]. CCF analysis showed that the correlation between the two signals was statistically significant (S1A Fig). This indicated that there was strong correlation between ERα36 signal (green) and mitochondrial signal (red).

To assess whether ERα36 was associated with cytoskeletal network, ht-UtSMC were subjected to double staining with ERα36 antibody and rhodamine phalloidin, a dye widely used to visualize F-actin in tissue sections, cell cultures, or cell-free preparations. Confocal images of the ht-UtSMC cells showed that ERα36 (green signal) and the phalloidin (red signal) stained different subcellular structures (Fig 2E–2G). The Pearson’s coefficient (r) between two channel signals was 0.267 (Fig 2H). There was no colocalization between ERα36 and F-actin in ht-UtSMC cells, based on the low coefficient value and the observation that ERα36 antibody and the rhodamine phalloidin stained independent structures within the cells. The ht-UtSMC cells were further analyzed with ERα36 antibody (red signal) and an endoplasmic reticulum marker, PDI (Fig 2I–2K). In ht-UtSMC cells, it appeared that ERα36 antibody and PDI stained distinctively different subcellular structures. The Pearson’s coefficient for the signal intensities of the two channels was 0.315 (Fig 2L), strongly suggesting there was no colocalization of ERα36 with endoplasmic reticulum.

**Fig 3:**
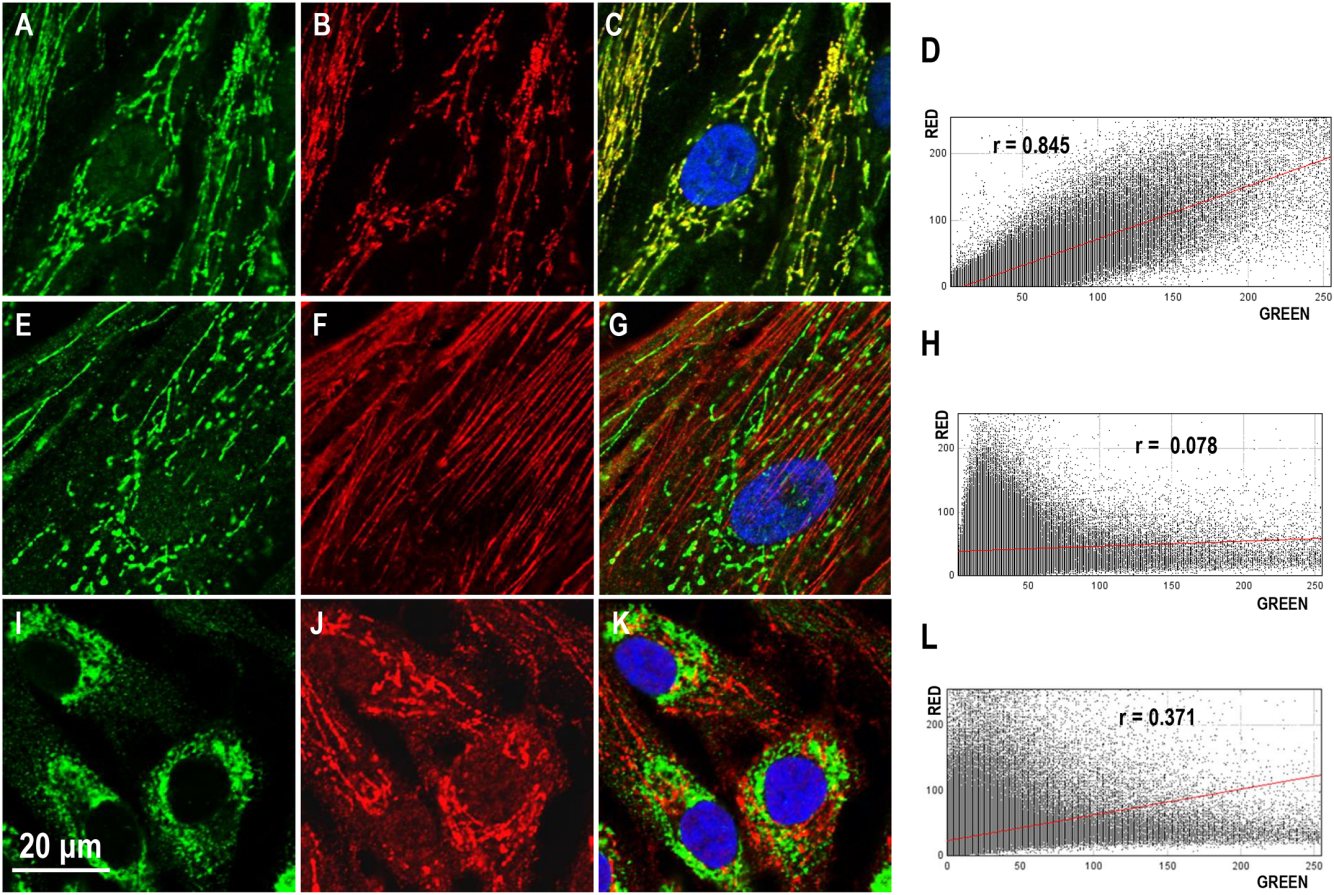
Colocalization analysis of ERα36 with mitochondria, F-actin or endoplasmic reticulum in ht-UtLM cells. Top row: A. ERα36 signal channel, B. MitoTracker signal, C. Merged image, D. Scatter plot; Middle row: E. ERα36 signal channel, F. F-actin dye signal, G. Merged image, H. Scatter plot; Bottom row: I. PDI signal channel, J. ERα36 signal channel, K. Merged image, L. Scatter plot. In the scatter plot, the symbol r represents the Pearson’s correlation coefficient. Scale bar = 20 μm for all images.

To determine if ERα36 had similar colocalization patterns with mitochondria, F-actin and endoplasmic reticulum in fibroid cells, we performed similar quantitative analysis for ht-UtLM cells (Fig 3A–3L). The colocalization pattern of ERα36 and MitoTracker were similar to that observed in ht-UtSMC cells (Fig 3A–3D). The Pearson's Coefficient for ht-UtLM cells was equal to 0.845 (Fig 3D). The CCF analysis indicated there was a significant drop in the value of the coefficient once the green channel image was shifted horizontally in either direction (S1B Fig). In ht-UtLM cells, the ERα36 (green) and rhodamine phalloidin (red) stained dissimilar subcellular structures (Fig 3E–3H), with the Pearson coefficient being equal to 0.078 (Fig 3H). Therefore, ERα36 was not associated with cytoskeletal F-actin filament. There was no signal colocalization between ERα36 (red) and endoplasmic reticulum marker PDI (green) (Fig 3I–3K), as the Pearson coefficient for the signal intensities was 0.371 (Fig 3L). These analyses strongly suggested that ERα36 was not localized in endoplasmic reticulum.

The above confocal and colocalization analyses demonstrated that ERα36 was co-localized with mitochondria, with almost no or minimal signal present in plasma membrane, nuclei, endoplasmic reticulum, or actin filaments in either non-cancerous cell line. We found similar findings in female reproductive tract cell lines such as human ovarian cancer (BG-1), breast cancer (MCF-7), endometrial cancer (Ishikawa) and vulvar leiomyosarcoma (SK-LMS-1) cells (S2 Fig).

### ERα36 peptide blocks ERα36 expression and mitochondrial colocalization

**Fig 4:**
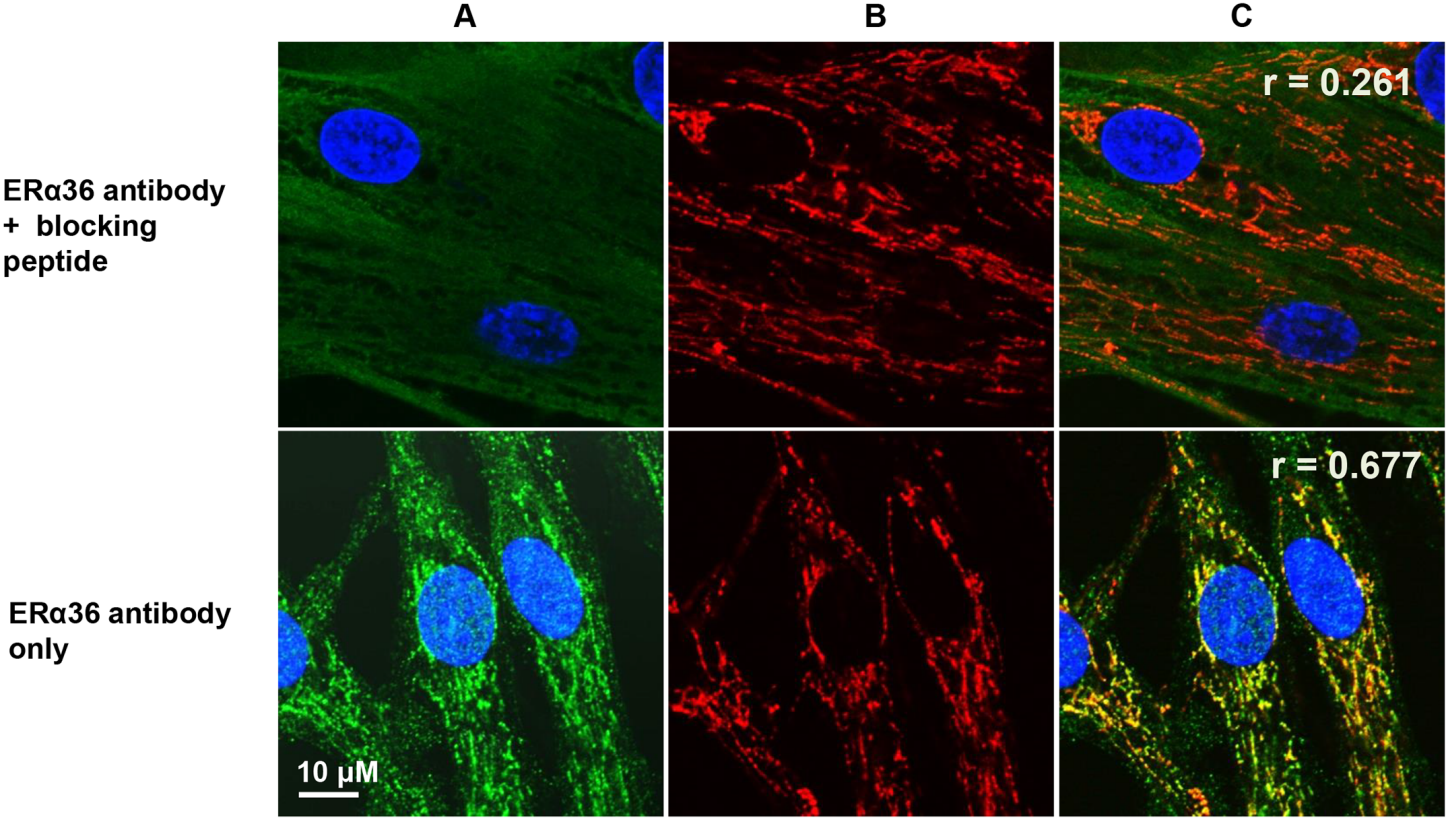
ERα36 blocking peptide competition assay in ht-UtLM cells. Ht-UtLM cells were incubated with MitoTracker and then stained with either neutralized ERα36 antibody (top row) or non-neutralized antibody (bottom row). Abbreviations: A. ERα36 signal; B. MitoTracker; C. Merged images. Scale bar = 10 μm for all images.

To determine whether the ERα36 signal was specifically due to the antibody binding to the unique ERα36 epitope, we did a competitive binding assay using a blocking peptide composed of the amino acid sequences unique to ERα36 (Fig 1). As shown in Fig 4, the neutralized ERα36 antibody failed to stain mitochondria, and the Pearson’s correlation coefficient between the two channels was 0.261. In contrast, the non-neutralized ERα36 antibody readily recognized mitochondrial structures with the coefficient being equal to 0.677. By comparing the staining pattern of the neutralized ERα36 antibody versus the antibody alone, we determined that the signal detected by ERα36 antibody in mitochondria was due to the unique ERα36 C-terminal peptide sequence (Fig 4).

### ERα36 association with subcellular membrane fractions

**Fig 5:**
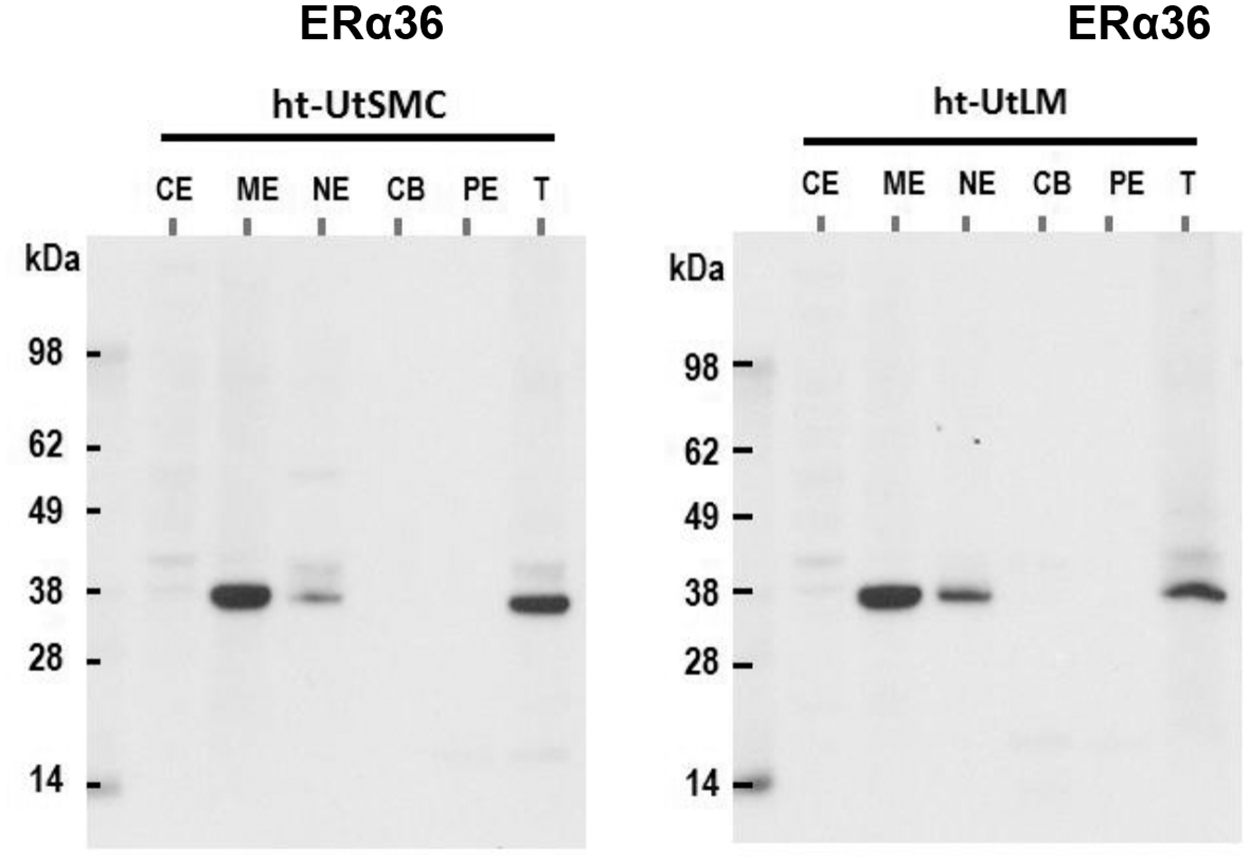
ERα36 Expression in subcellular fractions. Cellular Fraction Abbreviations: CE (cytoplasmic extract); ME (membrane extract); CB (chromatin-bound extract); NE (nuclear extract, nuclear soluble); PE (pellet extract, cytoskeleton); T (total cellular protein extract).

The subcellular fractionation process allows the stepwise separation of cytoplasmic, membrane, nuclear soluble, chromatin-bound, and cytoskeletal proteins. As shown in S3 Fig, the ht-UtSMC and ht-UtLM cells were fractionated into the five subcellular fractions. All the fractions were analyzed by the compartment specific antibodies such as HSP90 (cytoplasmic [cytosol] compartment-specific), EGFR (membrane-specific), SP1 (soluble nuclear fraction), H3 (chromatin-bound, nuclear insoluble), and Vimentin (cytoskeleton-specific). The western analysis showed that the two cell lines were well-fractionated as the antigen distribution was cellular compartment-specific (S3 Fig). When the fractionated samples were blotted with ERα36 antibody, only the membrane fraction displayed significant ERα36 signal at approximately 36 kDa (Fig 5). However, trace amounts of ERα36 were also detected in the nuclear soluble fractions for both cell types, which may due to the carryover between membrane and nuclear fractions; although minimal nuclear staining was also observed in the confocal images for both cell types.

We also isolated intact mitochondria from other cytosolic components in ht-UtSMC and ht-UtLM cells. To check for the purity of the isolated mitochondria, the preparations were blotted with antibodies against mitochondrial marker proteins such as mortalin, prohibitin, and Src [15–17]. When the fractionated mitochondrial preparations were blotted with mortalin antibodies, the signals were found exclusively in mitochondrial fractions of the two cell lines (S4A Fig). The Src signals were also significantly enriched in mitochondrial fractions although as to be expected, expression was also present in the cytosol (S4B Fig). As for prohibitin, we observed that the antigen was almost exclusively detected in the mitochondrial fractions (S4C Fig). These results suggested these three mitochondrial-specific proteins were abundantly and almost exclusively present in mitochondria.

### ERα36 is associated with mitochondrial specific protein, prohibitin

**Fig 6:**
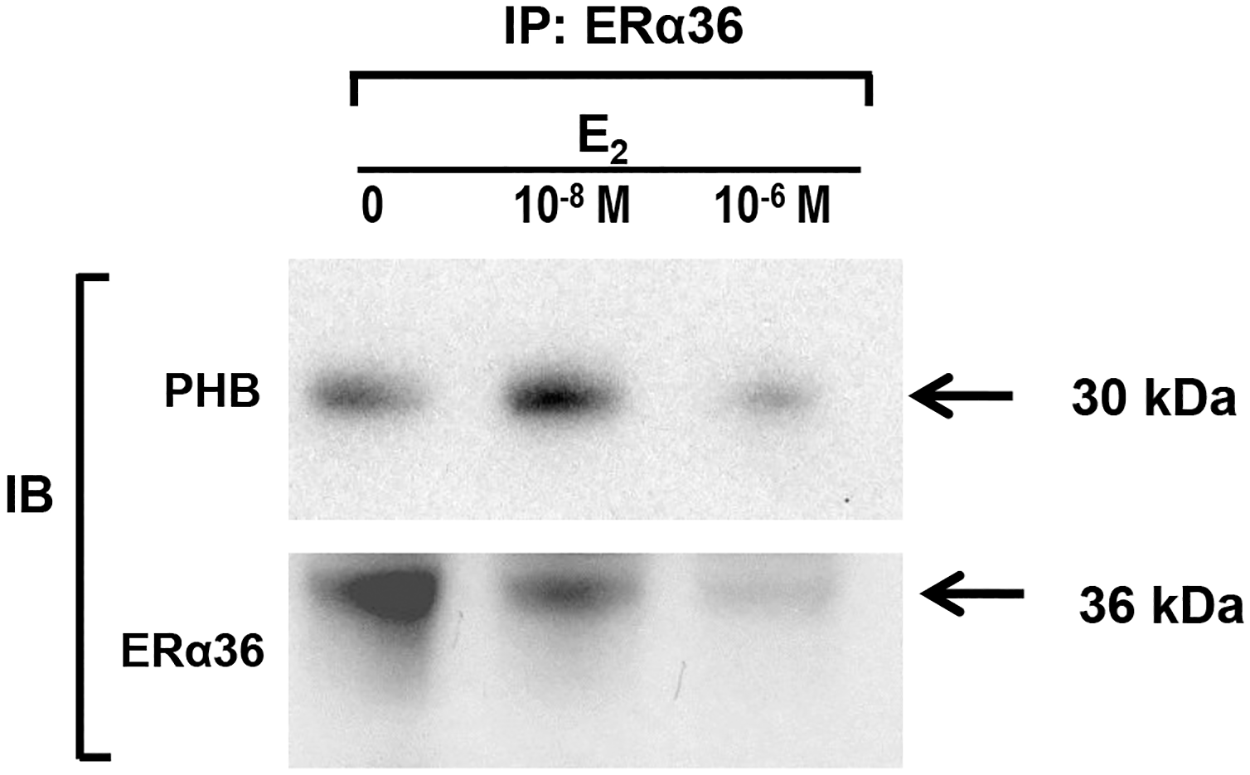
Interactions of ERα36 and prohibitin in human uterine leiomyoma cells. The interactions between ERα36 and prohibitin were determined with a co-immunoprecipitation (Co-IP). Ht-UtLM cells were treated with vehicle control, 10^−8^ M E_2_, and 10^−6^ M E_2_ for 24 hours. The blots presented are representative results that were repeated at least three times. PHB: prohibitin; IB: Immunobotting; IP: Immunoprecipitation.

It is known that the transcriptional activity of ERα is regulated by several coregulators, including prohibitin. Recently, the direct interaction of prohibitin-2 and the ERα ligand binding domain was demonstrated by structural analysis [16]. To determine whether ERα36 was associated with the mitochondrial protein prohibitin, interactions between ERα36 and prohibitin were evaluated with co-immunoprecipitation in ht-UtLM cells. As shown in Fig 5, the interactions were detected in control or samples treated with two concentrations (10^−8^ M or 10^−6^ M) of E_2_. The amount of prohibitin pulled down by ERα36 was the highest when the cultures were treated with 10^−8^ M E_2_ for 24 hours. Interestingly, the amount of ERα36 antigen detected was the highest when the cultures had not been subjected to E_2_ treatment, although association of ERα36 with prohibitin was less. It appeared that exogenous E_2_ decreased ERα36 expression in the cells, but increased its association with prohibitin at the lower concentration. The functional significance of the changes in ERα36 protein expression in response to E_2_ remains to be determined. However, the association of ERα36 with the mitochondrial-specific protein prohibitin was clearly visible (Fig 6).

## Discussion

Although there have been comprehensive reports on the subcellular localization of ERα36, non-genomic signaling and its role in malignant cancer cell growth [8–10], there are no studies, to our knowledge, that describe the distinctive staining pattern and specific mitochondrial subcellular localization of ERα36 observed in this study. A quantitative colocalization analysis with a Pearson’s correlation coefficient was applied to evaluate ERα36 subcellular localization in human uterine leiomyoma (ht-UtLM) and uterine smooth muscle (ht-UtSMC) cells. It is generally accepted that the fluorescence signals can be interpreted as colocalized when the Pearson’s correlation coefficient is greater than 0.5 [18,19]. With CCF analysis, colocalized structures show a peak at zero pixelshift and a bell-shaped curve, whereas, partially overlapping structures show a peak to one side of zero pixelshift [14]. Structures that are mutually exclusive of each other display a drop at zero pixelshift in CCF [14]. We studied ERα36 colocalization with various subcellular compartment markers targeting endoplasmic reticulum, a cytoskeletal filament, and mitochondria. In ht-UtSMC and ht-UtLM cells, the Pearson’s correlation coefficient between ERα36 and MitoTracker signal was equal to 0.826 and 0.845, respectively, indicating that ERα36 was significantly colocalized with mitochondria. With CCF analysis, both ht-UtSMC and ht-UtLM cells displayed a peak at zero pixelshift and symmetrical bell-shaped curves, further supporting the idea that ERα36 is colocalized with mitochondria [14]. On the other hand, the coefficients for F-actin and endoplasmic reticulum markers were significantly below the threshold required to be considered colocalized. Therefore, we excluded the possibility that ERα36 was associated with either F-actin or endoplasmic reticulum in these two cell lines to any great extent.

Two prohibitin homologues, Phb1 and Phb2, assemble into a high molecular weight complex of ~1.2 MDa in the mitochondrial inner membrane, and appear to be a reliable mitochondrial marker [20]. It is proposed that prohibitins serve as chaperones for respiration chain proteins or as general structural scaffolding required for optimal mitochondrial morphology and function [20]. The mitochondrial chaperone mortalin belongs to the Hsp70 family and is required for transporting protein from the cytoplasm to mitochondria [15]. Other proteins such as the Src family members (Fgr, Fyn, Lyn and c-Src) are all constitutively expressed and have been detected in mitochondria [21]. In our studies, western blot analysis with these mitochondrial markers suggested that the mitochondrial preparations were mostly free from contamination of other organelles and, in particular, prohibitin was specifically associated with mitochondria in ht-UtLM cells.

In addition to the fractionation studies, we found that ERα36 was co-precipitated with the mitochondrial-specific protein prohibitin. It is reported that prohibitin 2 binds to the ligand binding domain of ERα66 and represses its transcriptional activity [16]. It has been shown that E_2_ stimulates prohibitin expression in white adipose tissue and in the liver of rats [22]. In a cell culture model (3T3-L1 and C9 cells), transcription and protein levels of prohibitin were also dose-dependently increased by E_2_ treatment, further supporting *in vivo* data that prohibitin expression may be regulated by estrogens and intracellular steroid hormone signaling pathways [22]. We observed that 10^−8^ M E_2_ increased prohibitin protein expression and its association with ERα36, but reduced ERα36 levels in ht-UtLM cells. Also, a higher concentration of 10^−6^ M E_2_ reduced both prohibitin and ERα36 protein expression levels and their association in ht-UtLM cells. The difference in the responses to E_2_ observed in our study compared to that reported [22] may due to the unique cell types or concentrations of E_2_ used. The presence of ERα36 in mitochondria and its association with the mitochondrial protein prohibitin suggests that ERα36 may mediate critical roles in maintaining mitochondrial structure and function.

Immunofluorescence analyses of Ishikawa cancer cells have demonstrated that ERα36 is mostly present in the plasma membrane and cytoplasm, while ERα66 is predominantly expressed in the nucleus [23]. In Ishikawa cells, we also observed a similar ERα36 subcellular distribution pattern as previously reported [23]. In the four human female reproductive tract cancer cell lines (BG-1, Ishikawa, MCF-7, and SK-LMS-1) we evaluated, it appeared that the mitochondria were arranged in an extremely compact, perinuclear cytoplasmic space. As ERα36 is mostly associated with a mitochondrial network, the extreme compactness of the mitochondria may have contributed to the controversy associated with ERα36 subcellular localization in the literature [8–10].

Mitochondria are key cellular organelles that regulate critical processes such as energy production and apoptosis. Receptors for glucocorticoids, estrogens, androgens, and thyroid hormones have been detected in mitochondria of various cell types [24,25]. Both estrogen receptors ERα and ERβ have been found in the nucleus, plasma membrane, and in mitochondria, where they are proposed to mediate the differential physiological effects of estrogens [26,27]. It is suggested that estrogen receptors are imported into mitochondria, through tethering to cytosolic chaperone proteins and/or through direct interaction with mitochondrial import proteins [28]. Estrogen promotes mitochondrial activity by enhancing mitochondrial biogenesis and sustaining mitochondrial energy-producing capacity. Alterations in mitochondrial bioenergetic pathways is believed to mediate estrogen-induced tumorigenesis [29]. Estrogens have also been shown to affect concentrations and localization of anti-apoptotic factors, which appear to exert their anti-apoptotic effects via the maintenance of mitochondrial membrane potential in the face of stressors. Mitochondrial membrane potential collapse is a critical event leading to cell death, and the available data indicate that estrogens may protect mitochondria by preventing membrane potential collapse [28].

Due to ERα36’s unique domain architecture, its predominant mitochondrial presence, and the evidence that ERα36 is associated with the mitochondrial protein prohibitin in leiomyoma cells, we propose that ERα36 may be essential for maintaining mitochondrial structure and function. The potential interactions between ERα36 and the mitochondrial genome, as well as the interactions with mitochondrial-specific proteins critical for oxidative phosphorylation, oxidative stress protection, apoptosis, and estrogen signaling warrant further investigations. Additional studies on ERα36 and mitochondrial functions may shed light on human fibroid and cancer development mechanisms, and help to create effective treatment strategies in hormonally associated diseases, such as breast cancer. Targeting mitochondrial-associated ERα36 would be a promising therapeutic approach for the treatment of a variety of cancers and diseases, including fibroid tumors.

## Supporting information

S1 Fig Cross correlation function (CCF) analysis of the confocal images stained by ERα36 antibody and MitoTracker. A. ht-UtSMC cell culture. B. ht-UtLM cell culture. The CCF graphs were generated by Image J plugin JACoP.
(TIF)

S2 Fig ERα36 (green) and MitoTracker (red) colocalization analysis in BG-1, MCF-7, Ishikawa and SK-LMS-1 cell lines. DAPI, B. ERα36 signal, C. MitoTracker signal, D. Merged image.
(TIF)

S3 Fig Subcellular protein fractionations from human uterine cells analyzed with subcellular-specific markers. The subcellular fractionation procedure was effective in separating the subcellular components, as shown by western blot analyses with respective subcellular markers. Abbreviations: HSP90 (HSP90 Rabbit mAb, Cell Signaling #4877), EGFR (EGFR Rabbit Polyclonal Antibody, Santa Cruz Biotechnology Cat# sc-03), SP1 (SP1 Rabbit mAb, Cell Signaling Cat# 9389), H3 (Histone H3 Rabbit Polyclonal Antibody, Cell Signaling Cat#9715), Vimentin (Vimentin Rabbit polyclonal Antibody, Cell Signaling Cat# 3932). CE (cytoplasmic extract), ME (membrane extract), NE (nuclear extract, nuclear soluble), CB (chromatin-bound extract), PE (pellet extract, cytoskeleton).
(TIF)

S4 Fig Western blot analysis of mitochondrial fractions with specific mitochondrial markers. (A) Blot is probed for Mortalin expression. (B) Blot was probed for Src expression. (C). Blot was probed for Prohibitin expression. Abbreviations: C. Cytosol fraction; M. Mitochondrial fraction.
(TIF)
