# Supplementary figures and images for: ERα36, a variant of estrogen receptor α, is predominantly localized in mitochondria of human uterine smooth muscle and leiomyoma cells
Source: PLoS One. 2017 Oct 11;12(10):e0186078. doi: 10.1371/journal.pone.0186078 (PMC5636123; doi:10.1371/journal.pone.0186078)

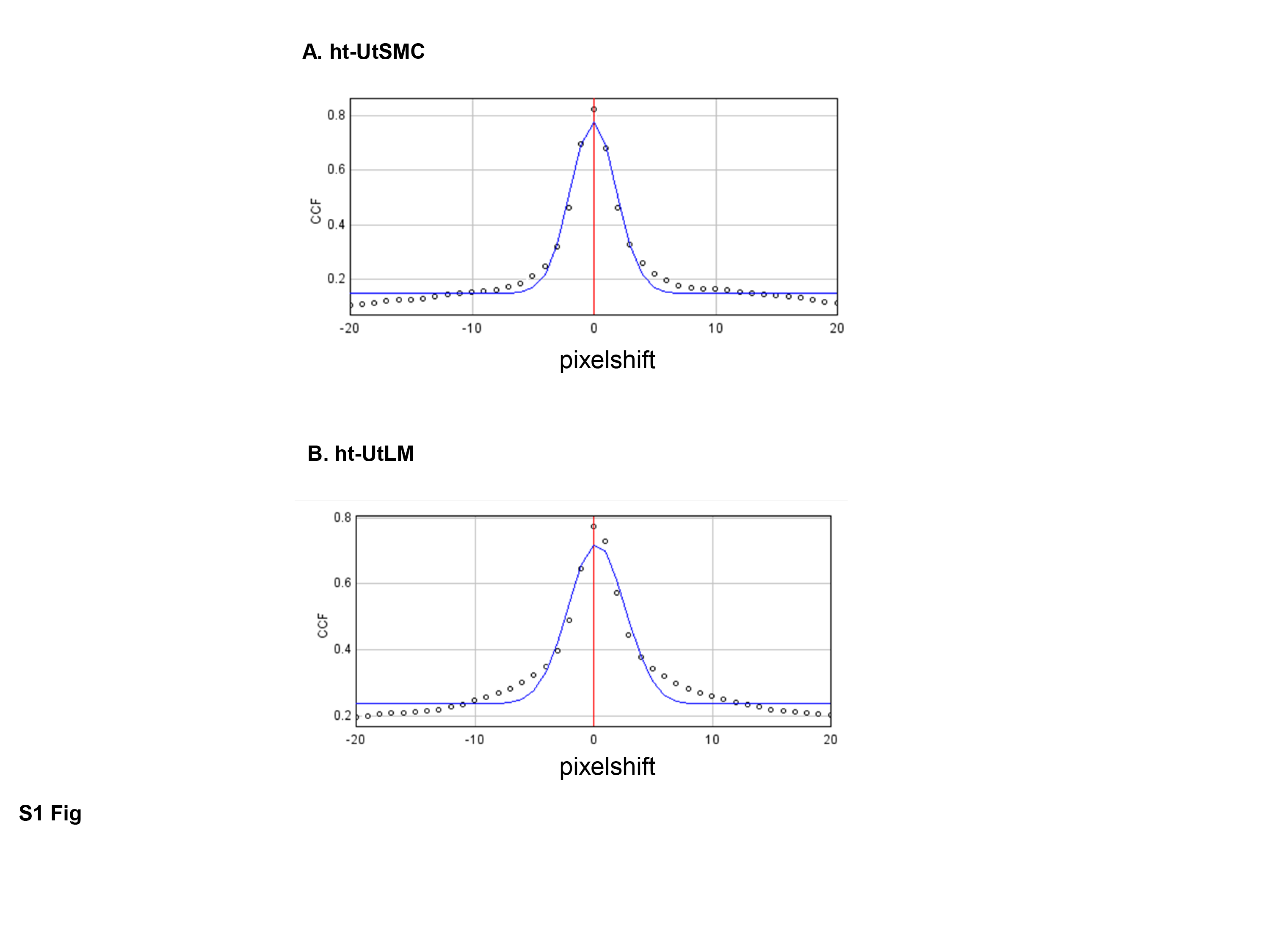

Supplement: S1 Fig — A. ht-UtSMC cell culture. B. ht-UtLM cell culture. The CCF graphs were generated by Image J plugin JACoP. (TIF) [file pone.0186078.s001.tif]

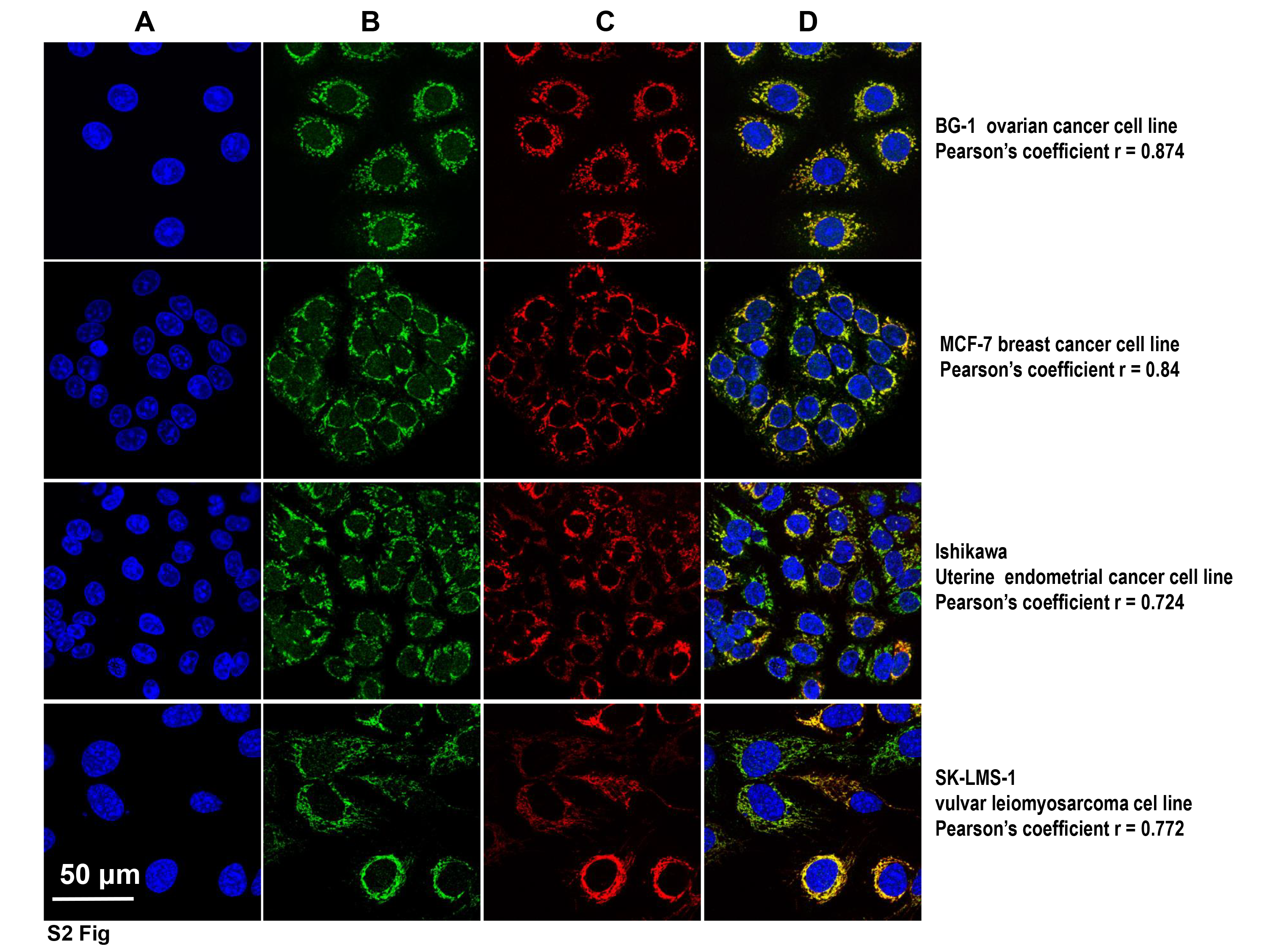

Supplement: S2 Fig — DAPI, B. ERα36 signal, C. MitoTracker signal, D. Merged image. (TIF) [file pone.0186078.s002.tif]

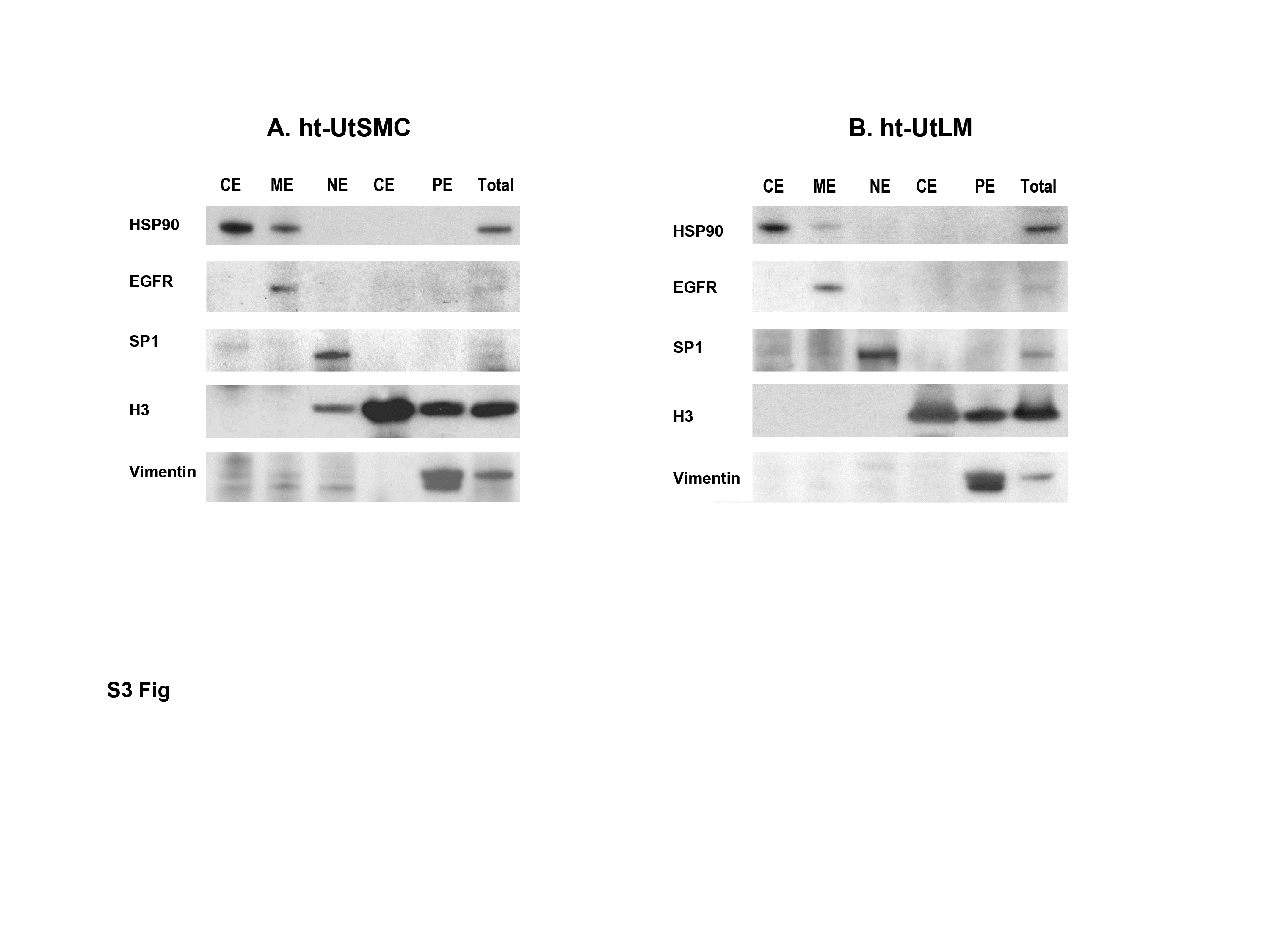

Supplement: S3 Fig — The subcellular fractionation procedure was effective in separating the subcellular components, as shown by western blot analyses with respective subcellular markers. Abbreviations: HSP90 (HSP90 Rabbit mAb, Cell Signaling #4877), EGFR (EGFR Rabbit Polyclonal Antibody, Santa Cruz Biotechnology Cat# sc-03), SP1 (SP1 Rabbit mAb, Cell Signaling Cat# 9389), H3 (Histone H3 Rabbit Polyclonal Antibody, Cell Signaling Cat#9715), Vimentin (Vimentin Rabbit polyclonal Antibody, Cell Signaling Cat# 3932). CE (cytoplasmic extract), ME (membrane extract), NE (nuclear extract, nuclear soluble), CB (chromatin-bound extract), PE (pellet extract, cytoskeleton). (TIF) [file pone.0186078.s003.tif]

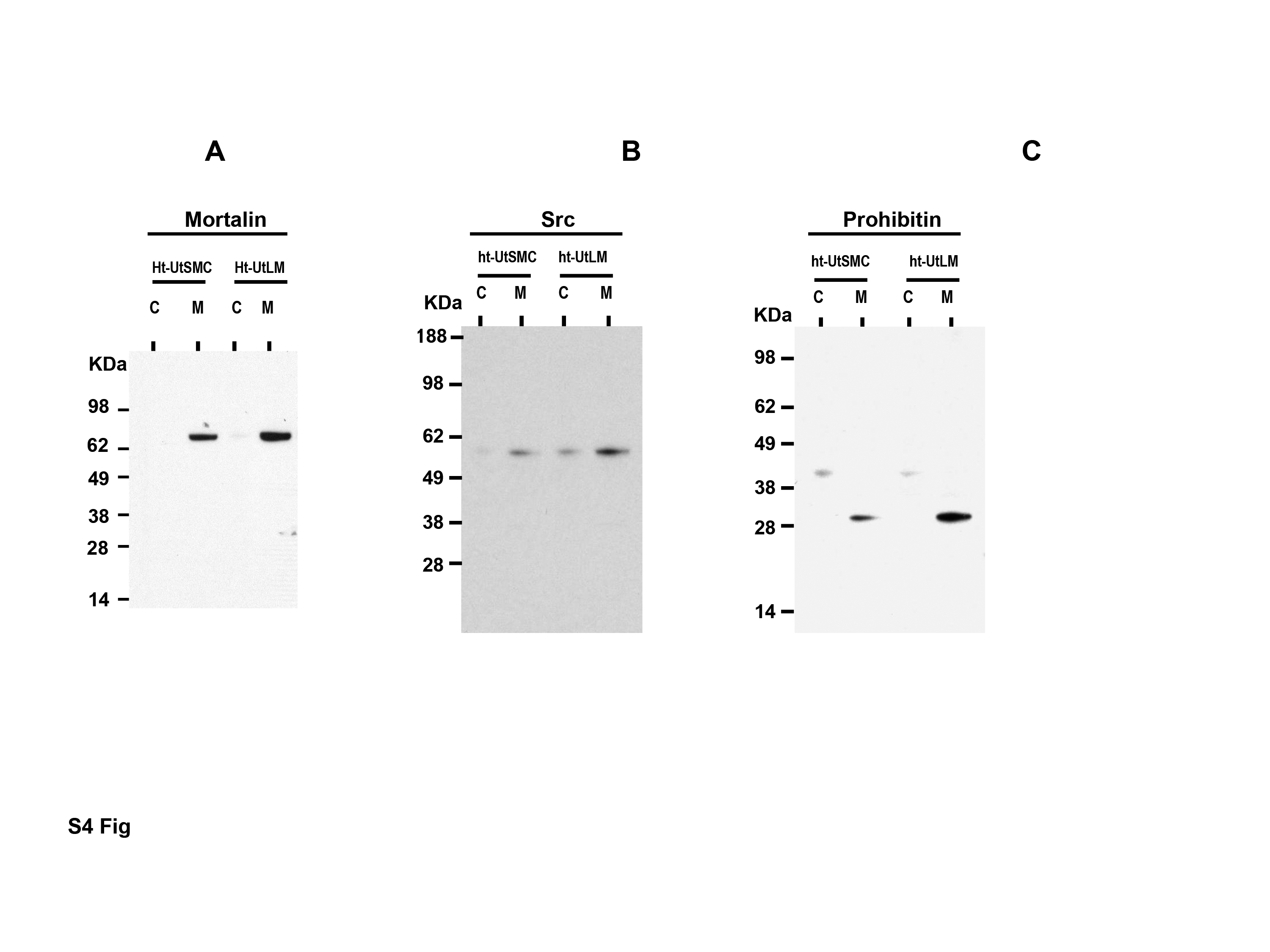

Supplement: S4 Fig — (A) Blot is probed for Mortalin expression. (B) Blot was probed for Src expression. (C). Blot was probed for Prohibitin expression. Abbreviations: C. Cytosol fraction; M. Mitochondrial fraction. (TIF) [file pone.0186078.s004.tif]
